# Diaqua­[3,5-bis­(4-pyrid­yl)-1*H*-1,2,4-triazole-κ*N* ^3^](pyridine-2,6-dicarboxyl­ato-κ^3^ *O* ^2^,*N*,*O* ^6^)nickel(II)

**DOI:** 10.1107/S1600536810053687

**Published:** 2011-01-08

**Authors:** Feng-Jie Cheng, Yi-Sen Wang, Yan-Cheng Liu

**Affiliations:** a Key Laboratory for the Chemistry and Molecular Engineering of Medicinal Resources (Ministry of Education of China), School of Chemistry & Chemical Engineering of Guangxi Normal University, Guilin 541004, People’s Republic of China; b Petrochemical Research Institute of PetroChina Co. Ltd, Beijing 100195, People’s Republic of China

## Abstract

In the title compound, [Ni(C_7_H_3_NO_4_)(C_12_H_9_N_5_)(H_2_O)_2_], the Ni^II^ atom is coordinated in a distorted octa­hedral geometry by one N and two O atoms from a pyridine-2,6-dicarboxyl­ate ligand, one N atom from a 3,5-bis­(4-pyrid­yl)-1*H*-1,2,4-triazole ligand in equatorial positions and two water mol­ecules in axial positions. The crystal packing is consolidated by inter­molecular O—H⋯O, O—H⋯N and N—H⋯O hydrogen bonds.

## Related literature

For synthesis of the 1*H*-3,5-bis­(4-pyrid­yl)-1,2,4-triazole (BPT) ligand, see: Liu *et al.* (2004 ▶). For BPT–metal complexes, see: Huang *et al.* (2010*a* ▶,*b* ▶).
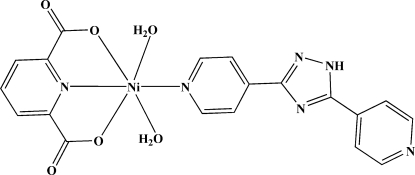


## Experimental

### 

#### Crystal data

[Ni(C_7_H_3_NO_4_)(C_12_H_9_N_5_)(H_2_O)_2_]
*M* *_r_* = 483.07
Monoclinic, 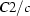

*a* = 26.434 (5) Å
*b* = 6.0190 (12) Å
*c* = 26.170 (5) Å
β = 105.69 (3)°
*V* = 4008.7 (13) Å^3^
*Z* = 8
Mo *K*α radiation
μ = 1.02 mm^−1^
*T* = 293 K
0.32 × 0.21 × 0.11 mm

#### Data collection

Bruker SMART CCD area-detector diffractometer
Absorption correction: multi-scan (*SADABS*; Sheldrick, 1996 ▶) *T*_min_ = 0.768, *T*_max_ = 0.910
10662 measured reflections
3534 independent reflections
2669 reflections with *I* > 2σ(*I*)
*R*_int_ = 0.071

#### Refinement

*R*[*F* ^2^ > 2σ(*F* ^2^)] = 0.063
*wR*(*F* ^2^) = 0.104
*S* = 1.11
3534 reflections
310 parameters
7 restraints
H atoms treated by a mixture of independent and constrained refinement
Δρ_max_ = 0.33 e Å^−3^
Δρ_min_ = −0.33 e Å^−3^

### 

Data collection: *SMART* (Bruker, 2007 ▶); cell refinement: *SAINT* (Bruker, 2007 ▶); data reduction: *SAINT*; program(s) used to solve structure: *SHELXS97* (Sheldrick, 2008 ▶); program(s) used to refine structure: *SHELXL97* (Sheldrick, 2008 ▶); molecular graphics: *PLATON* (Spek, 2009 ▶); software used to prepare material for publication: *SHELXL97*.

## Supplementary Material

Crystal structure: contains datablocks I, global. DOI: 10.1107/S1600536810053687/cv5017sup1.cif

Structure factors: contains datablocks I. DOI: 10.1107/S1600536810053687/cv5017Isup2.hkl

Additional supplementary materials:  crystallographic information; 3D view; checkCIF report

## Figures and Tables

**Table 1:** Hydrogen-bond geometry (Å, °)

*D*—H⋯*A*	*D*—H	H⋯*A*	*D*⋯*A*	*D*—H⋯*A*
N5—H5⋯O4^i^	0.88 (4)	1.81 (4)	2.685 (5)	179 (7)
O5—H5*B*⋯O1^ii^	0.86 (4)	1.87 (4)	2.718 (5)	168 (5)
O5—H5*C*⋯O2^iii^	0.86 (4)	1.89 (4)	2.705 (5)	160 (4)
O6—H6*B*⋯N6^iv^	0.87 (4)	1.93 (4)	2.781 (5)	167 (6)
O6—H6*C*⋯O3^v^	0.86 (4)	2.39 (4)	3.246 (5)	169 (8)
O6—H6*C*⋯O4^v^	0.86 (4)	2.41 (7)	3.081 (5)	135 (6)

Symmetry codes: (i) 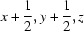; (ii) 
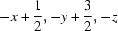
; (iii) 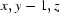; (iv) 
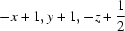
; (v) 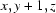.

**Fig. 1.**
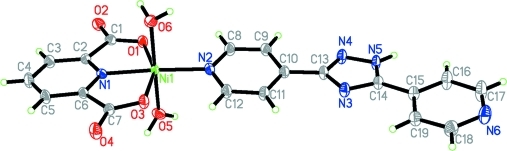
The molecular structure of the title compound showing the atom-labelling scheme. Displacement ellipsoids are drawn at the 30% probability level.

## supplementary crystallographic information

### Comment

As a contribution to structural studies of coordination abilities of 1*H*-3,5-bis(4-pyridyl)-1,2,4-triazole (BPT) ligand (Huang *et al.*, 2010*a*,b), we present here the crystal structure of the title compound (Fig. 1)- a new nickel(II) complex with the BPT and pyridine-2,6-dicarboxylate(PDC) ligands.

Within the title compound, the nickel(II) center is six-coordinated respectively by two carboxylic O atoms [Ni1—O1 2.112 (3) Å, Ni1—O3 2.103 (3) Å,] and one N atom of PDC [Ni1—N1 1.989 (3) Å], as well as one N atom of BPT ligand [Ni1—N2 2.054 (3) Å], from the equatorial orientation, while two O atoms of two H_2_O occupy the axial positions. Nickel(II) center shows a distorted octahedral coordination geometries, with bond angles of N1—Ni1—N2 175.31 (14)°, O1—Ni1—O3 156.12 (10) ° and O5—Ni1—O6 176.93 (14)°, respectively. Viewed from the whole crystal structure, each complex is linked together by intermolecular O—H···O hydrogen bonds from H_2_O and carboxyl of PDC to form a supramolecular structure.

### Experimental

1*H*-3,5-bis(4-pyridyl)-1,2,4-triazole (BPT) was prepared according to the literature (Liu *et al.*, 2004). A mixture of pyridine-2,6-dicarboxylic acid (0.084 g, 0.5 mmol), Ni(NO_3_)_2_.6H_2_O (0.145 g, 0.5 mmol), NaOH (0.040 g, 1 mmol), BPT (0.112 g, 0.5 mmol), ethanol (0.1 ml), and H_2_O (10 ml) was enclosed in a Teflon-lined autoclave under autogenous pressure at 383 K for 4 days after stirring. Red block crystals were then obtained with yield of 47% (based on Ni^II^). Single crystals fit for X-ray diffraction analysis was picked out for measurement. Elemental analysis calculated for C_19_H_16_N_6_NiO_6_: C, 47.20; H, 3.31; N, 17.39. Found: C, 46.97; H, 3.51; N, 17.21.

### Refinement

C-bound H atoms were geometrically positioned (C—H 0.93 Å) and allowed to ride on their parent atoms, with *U*_iso_(H) = 1.2 *U*_eq_(C). N– and O-bound H atoms were located on a difference map and refined isotropically with restraints (O—H = 0.85 (4) Å; N—H = 0.90 (4) Å).

### Crystal data

**Table:**

[Ni(C_7_H_3_NO_4_)(C_12_H_9_N_5_)(H_2_O)_2_]	*F*(000) = 1984
*M**_r_* = 483.07	*D*_x_ = 1.601 Mg m^−^^3^
Monoclinic, *C*2/*c*	Mo *K*α radiation, λ = 0.71073 Å
*a* = 26.434 (5) Å	θ = 1.5–25.1°
*b* = 6.0190 (12) Å	µ = 1.02 mm^−^^1^
*c* = 26.170 (5) Å	*T* = 293 K
β = 105.69 (3)°	Block, green
*V* = 4008.7 (13) Å^3^	0.32 × 0.21 × 0.11 mm
*Z* = 8	

### Data collection

**Table:**

Bruker SMART CCD area-detector diffractometer	3534 independent reflections
Radiation source: fine-focus sealed tube	2669 reflections with *I* > 2σ(*I*)
graphite	*R*_int_ = 0.071
phi and ω scans	θ_max_ = 25.1°, θ_min_ = 3.2°
Absorption correction: multi-scan (*SADABS*; Sheldrick, 1996)	*h* = −31→31
*T*_min_ = 0.768, *T*_max_ = 0.910	*k* = −6→7
10662 measured reflections	*l* = −31→30

### Refinement

**Table:**

Refinement on *F*^2^	Secondary atom site location: difference Fourier map
Least-squares matrix: full	Hydrogen site location: inferred from neighbouring sites
*R*[*F*^2^ > 2σ(*F*^2^)] = 0.063	H atoms treated by a mixture of independent and constrained refinement
*wR*(*F*^2^) = 0.104	*w* = 1/[σ^2^(*F*_o_^2^) + (0.0251*P*)^2^ + 7.5822*P*] where *P* = (*F*_o_^2^ + 2*F*_c_^2^)/3
*S* = 1.11	(Δ/σ)_max_ < 0.001
3534 reflections	Δρ_max_ = 0.33 e Å^−^^3^
310 parameters	Δρ_min_ = −0.33 e Å^−^^3^
7 restraints	Extinction correction: *SHELXL97* (Sheldrick, 2008), Fc^*^=kFc[1+0.001xFc^2^λ^3^/sin(2θ)]^-1/4^
Primary atom site location: structure-invariant direct methods	Extinction coefficient: 0.00023 (7)

### Special details

**Table:**

Geometry. All esds (except the esd in the dihedral angle between two l.s. planes) are estimated using the full covariance matrix. The cell esds are taken into account individually in the estimation of esds in distances, angles and torsion angles; correlations between esds in cell parameters are only used when they are defined by crystal symmetry. An approximate (isotropic) treatment of cell esds is used for estimating esds involving l.s. planes.
Refinement. Refinement of *F*^2^ against ALL reflections. The weighted *R*-factor *wR* and goodness of fit *S* are based on *F*^2^, conventional *R*-factors *R* are based on *F*, with *F* set to zero for negative *F*^2^. The threshold expression of *F*^2^ > σ(*F*^2^) is used only for calculating *R*-factors(gt) *etc*. and is not relevant to the choice of reflections for refinement. *R*-factors based on *F*^2^ are statistically about twice as large as those based on *F*, and *R*- factors based on ALL data will be even larger.

### Fractional atomic coordinates and isotropic or equivalent isotropic displacement parameters (Å^2^)

**Table:**

	*x*	*y*	*z*	*U*_iso_*/*U*_eq_	
Ni1	0.226457 (19)	0.64878 (9)	0.08540 (2)	0.02533 (18)	
C1	0.16122 (16)	0.9930 (7)	0.02297 (17)	0.0284 (10)	
C2	0.12404 (14)	0.8314 (7)	0.03879 (16)	0.0246 (9)	
C3	0.06954 (15)	0.8470 (7)	0.02545 (18)	0.0350 (11)	
H3A	0.0521	0.9624	0.0043	0.042*	
C4	0.04206 (16)	0.6862 (8)	0.04454 (19)	0.0412 (12)	
H4A	0.0056	0.6929	0.0363	0.049*	
C5	0.06859 (15)	0.5162 (7)	0.07569 (18)	0.0341 (11)	
H5A	0.0504	0.4075	0.0887	0.041*	
C6	0.12286 (14)	0.5098 (7)	0.08726 (16)	0.0240 (10)	
C7	0.16017 (15)	0.3376 (7)	0.11997 (16)	0.0277 (10)	
C8	0.34025 (16)	0.7559 (7)	0.09586 (18)	0.0354 (11)	
H8A	0.3270	0.8909	0.0807	0.042*	
C9	0.39403 (16)	0.7200 (7)	0.10786 (18)	0.0334 (11)	
H9A	0.4161	0.8294	0.1009	0.040*	
C10	0.41453 (15)	0.5203 (7)	0.13023 (17)	0.0296 (10)	
C11	0.37953 (15)	0.3656 (7)	0.13924 (18)	0.0364 (11)	
H11A	0.3916	0.2291	0.1543	0.044*	
C12	0.32645 (16)	0.4132 (7)	0.12595 (19)	0.0361 (12)	
H12A	0.3036	0.3052	0.1320	0.043*	
C13	0.47160 (15)	0.4758 (7)	0.14512 (17)	0.0299 (10)	
C14	0.54546 (15)	0.3328 (8)	0.17986 (16)	0.0302 (10)	
C15	0.58930 (16)	0.1971 (7)	0.21043 (17)	0.0318 (11)	
C16	0.64134 (17)	0.2613 (8)	0.2184 (2)	0.0485 (14)	
H16A	0.6497	0.3918	0.2035	0.058*	
C17	0.68063 (18)	0.1269 (9)	0.2492 (2)	0.0579 (16)	
H17A	0.7153	0.1726	0.2544	0.070*	
C18	0.62206 (18)	−0.1208 (8)	0.26260 (19)	0.0441 (13)	
H18A	0.6149	−0.2535	0.2774	0.053*	
C19	0.57996 (17)	0.0004 (8)	0.23279 (18)	0.0388 (12)	
H19A	0.5458	−0.0507	0.2280	0.047*	
N1	0.14859 (11)	0.6669 (5)	0.06894 (13)	0.0231 (8)	
N2	0.30646 (12)	0.6050 (6)	0.10502 (14)	0.0282 (9)	
N3	0.49458 (12)	0.3023 (6)	0.17511 (14)	0.0329 (9)	
N4	0.50503 (12)	0.6110 (6)	0.13012 (15)	0.0363 (10)	
N5	0.55201 (13)	0.5164 (6)	0.15348 (15)	0.0352 (10)	
N6	0.67239 (15)	−0.0606 (7)	0.27162 (16)	0.0473 (11)	
O1	0.20996 (10)	0.9403 (4)	0.03895 (11)	0.0304 (7)	
O2	0.14263 (11)	1.1605 (5)	−0.00256 (12)	0.0382 (8)	
O3	0.20889 (10)	0.3681 (5)	0.12533 (11)	0.0312 (7)	
O4	0.14157 (11)	0.1758 (5)	0.13800 (13)	0.0427 (8)	
O5	0.21976 (13)	0.4697 (5)	0.01727 (13)	0.0394 (8)	
O6	0.23372 (12)	0.8463 (6)	0.15262 (13)	0.0394 (8)	
H5C	0.1995 (14)	0.360 (5)	0.0075 (17)	0.055 (16)*	
H6B	0.2635 (11)	0.861 (8)	0.1761 (15)	0.070 (19)*	
H5B	0.2419 (14)	0.479 (7)	−0.0008 (16)	0.060 (17)*	
H5	0.5810 (13)	0.575 (8)	0.147 (2)	0.076 (19)*	
H6C	0.222 (2)	0.977 (5)	0.145 (3)	0.17 (4)*	

### Atomic displacement parameters (Å^2^)

**Table:**

	*U*^11^	*U*^22^	*U*^33^	*U*^12^	*U*^13^	*U*^23^
Ni1	0.0181 (3)	0.0257 (3)	0.0334 (3)	−0.0014 (3)	0.0092 (2)	0.0005 (3)
C1	0.030 (3)	0.029 (3)	0.024 (3)	−0.004 (2)	0.004 (2)	−0.003 (2)
C2	0.024 (2)	0.023 (2)	0.027 (2)	−0.0006 (19)	0.0068 (19)	−0.001 (2)
C3	0.026 (2)	0.029 (2)	0.046 (3)	0.003 (2)	0.003 (2)	0.007 (2)
C4	0.020 (2)	0.044 (3)	0.059 (3)	0.001 (2)	0.010 (2)	0.007 (3)
C5	0.024 (2)	0.033 (3)	0.048 (3)	−0.006 (2)	0.013 (2)	0.010 (2)
C6	0.021 (2)	0.025 (2)	0.027 (2)	−0.0019 (18)	0.0075 (19)	0.001 (2)
C7	0.028 (2)	0.027 (2)	0.030 (2)	−0.002 (2)	0.011 (2)	−0.001 (2)
C8	0.027 (2)	0.032 (3)	0.045 (3)	0.002 (2)	0.005 (2)	0.009 (2)
C9	0.027 (2)	0.029 (3)	0.046 (3)	−0.0044 (19)	0.013 (2)	0.007 (2)
C10	0.023 (2)	0.032 (3)	0.034 (3)	−0.0009 (19)	0.008 (2)	0.000 (2)
C11	0.026 (2)	0.030 (3)	0.056 (3)	0.003 (2)	0.014 (2)	0.012 (2)
C12	0.023 (2)	0.033 (3)	0.054 (3)	−0.001 (2)	0.012 (2)	0.010 (2)
C13	0.020 (2)	0.038 (3)	0.032 (3)	0.002 (2)	0.007 (2)	0.008 (2)
C14	0.022 (2)	0.037 (3)	0.032 (3)	0.003 (2)	0.007 (2)	0.010 (2)
C15	0.027 (2)	0.040 (3)	0.029 (3)	0.003 (2)	0.009 (2)	0.007 (2)
C16	0.026 (3)	0.055 (3)	0.064 (4)	0.005 (2)	0.013 (3)	0.034 (3)
C17	0.027 (3)	0.072 (4)	0.073 (4)	0.007 (3)	0.011 (3)	0.033 (3)
C18	0.044 (3)	0.042 (3)	0.044 (3)	0.004 (2)	0.009 (3)	0.012 (3)
C19	0.030 (3)	0.044 (3)	0.041 (3)	0.001 (2)	0.006 (2)	0.007 (2)
N1	0.0214 (18)	0.0195 (18)	0.0280 (19)	−0.0029 (16)	0.0057 (16)	−0.0002 (17)
N2	0.0193 (18)	0.031 (2)	0.033 (2)	−0.0017 (16)	0.0054 (16)	−0.0002 (17)
N3	0.0222 (19)	0.038 (2)	0.040 (2)	0.0020 (17)	0.0104 (18)	0.0104 (19)
N4	0.0206 (19)	0.038 (2)	0.052 (3)	0.0041 (17)	0.0113 (18)	0.015 (2)
N5	0.017 (2)	0.039 (2)	0.049 (3)	0.0041 (17)	0.0074 (19)	0.014 (2)
N6	0.035 (2)	0.057 (3)	0.047 (3)	0.012 (2)	0.005 (2)	0.021 (2)
O1	0.0237 (16)	0.0296 (17)	0.0403 (19)	−0.0028 (13)	0.0129 (14)	0.0045 (15)
O2	0.0390 (18)	0.0273 (17)	0.0454 (19)	−0.0032 (15)	0.0063 (15)	0.0097 (17)
O3	0.0206 (15)	0.0348 (17)	0.0394 (18)	0.0019 (14)	0.0103 (14)	0.0095 (15)
O4	0.0305 (17)	0.0379 (19)	0.061 (2)	−0.0006 (15)	0.0153 (16)	0.0213 (18)
O5	0.044 (2)	0.036 (2)	0.046 (2)	−0.0169 (17)	0.0237 (18)	−0.0124 (17)
O6	0.0326 (19)	0.044 (2)	0.036 (2)	0.0048 (17)	−0.0016 (16)	−0.0132 (18)

### Geometric parameters (Å, °)

**Table:**

Ni1—N1	1.989 (3)	C10—C13	1.477 (5)
Ni1—O5	2.050 (3)	C11—C12	1.381 (5)
Ni1—N2	2.054 (3)	C11—H11A	0.9300
Ni1—O6	2.088 (3)	C12—N2	1.325 (5)
Ni1—O3	2.103 (3)	C12—H12A	0.9300
Ni1—O1	2.112 (3)	C13—N4	1.336 (5)
C1—O2	1.236 (5)	C13—N3	1.348 (5)
C1—O1	1.282 (4)	C14—N3	1.330 (5)
C1—C2	1.518 (5)	C14—N5	1.339 (5)
C2—N1	1.322 (5)	C14—C15	1.466 (6)
C2—C3	1.391 (5)	C15—C19	1.372 (6)
C3—C4	1.382 (6)	C15—C16	1.389 (6)
C3—H3A	0.9300	C16—C17	1.389 (6)
C4—C5	1.376 (6)	C16—H16A	0.9300
C4—H4A	0.9300	C17—N6	1.317 (6)
C5—C6	1.384 (5)	C17—H17A	0.9300
C5—H5A	0.9300	C18—N6	1.337 (5)
C6—N1	1.328 (5)	C18—C19	1.382 (6)
C6—C7	1.524 (6)	C18—H18A	0.9300
C7—O4	1.241 (5)	C19—H19A	0.9300
C7—O3	1.271 (4)	N4—N5	1.352 (4)
C8—N2	1.340 (5)	N5—H5	0.901 (19)
C8—C9	1.387 (5)	O5—H5C	0.842 (18)
C8—H8A	0.9300	O5—H5B	0.848 (18)
C9—C10	1.382 (6)	O6—H6B	0.861 (19)
C9—H9A	0.9300	O6—H6C	0.854 (19)
C10—C11	1.377 (5)		
			
N1—Ni1—O5	89.97 (13)	C10—C11—H11A	120.0
N1—Ni1—N2	175.33 (14)	C12—C11—H11A	120.0
O5—Ni1—N2	89.48 (14)	N2—C12—C11	123.3 (4)
N1—Ni1—O6	90.30 (13)	N2—C12—H12A	118.3
O5—Ni1—O6	177.00 (14)	C11—C12—H12A	118.3
N2—Ni1—O6	90.49 (13)	N4—C13—N3	114.4 (3)
N1—Ni1—O3	78.29 (12)	N4—C13—C10	121.4 (4)
O5—Ni1—O3	91.78 (12)	N3—C13—C10	124.2 (4)
N2—Ni1—O3	97.09 (12)	N3—C14—N5	109.5 (4)
O6—Ni1—O3	91.20 (13)	N3—C14—C15	127.2 (4)
N1—Ni1—O1	77.83 (12)	N5—C14—C15	123.2 (4)
O5—Ni1—O1	88.88 (13)	C19—C15—C16	117.4 (4)
N2—Ni1—O1	106.79 (12)	C19—C15—C14	120.4 (4)
O6—Ni1—O1	88.25 (13)	C16—C15—C14	122.1 (4)
O3—Ni1—O1	156.11 (10)	C15—C16—C17	118.7 (4)
O2—C1—O1	126.6 (4)	C15—C16—H16A	120.7
O2—C1—C2	118.6 (4)	C17—C16—H16A	120.7
O1—C1—C2	114.9 (4)	N6—C17—C16	124.7 (4)
N1—C2—C3	120.6 (4)	N6—C17—H17A	117.6
N1—C2—C1	113.2 (3)	C16—C17—H17A	117.6
C3—C2—C1	126.3 (4)	N6—C18—C19	124.6 (5)
C4—C3—C2	118.2 (4)	N6—C18—H18A	117.7
C4—C3—H3A	120.9	C19—C18—H18A	117.7
C2—C3—H3A	120.9	C15—C19—C18	119.1 (4)
C5—C4—C3	120.1 (4)	C15—C19—H19A	120.5
C5—C4—H4A	120.0	C18—C19—H19A	120.5
C3—C4—H4A	120.0	C2—N1—C6	122.2 (3)
C4—C5—C6	118.8 (4)	C2—N1—Ni1	118.9 (3)
C4—C5—H5A	120.6	C6—N1—Ni1	118.9 (3)
C6—C5—H5A	120.6	C12—N2—C8	117.0 (3)
N1—C6—C5	120.2 (4)	C12—N2—Ni1	118.9 (3)
N1—C6—C7	111.7 (3)	C8—N2—Ni1	124.0 (3)
C5—C6—C7	128.1 (4)	C14—N3—C13	103.5 (3)
O4—C7—O3	124.5 (4)	C13—N4—N5	102.2 (3)
O4—C7—C6	119.0 (3)	C14—N5—N4	110.5 (3)
O3—C7—C6	116.5 (4)	C14—N5—H5	131 (3)
N2—C8—C9	123.1 (4)	N4—N5—H5	118 (3)
N2—C8—H8A	118.5	C17—N6—C18	115.5 (4)
C9—C8—H8A	118.5	C1—O1—Ni1	115.2 (2)
C10—C9—C8	119.5 (4)	C7—O3—Ni1	114.5 (3)
C10—C9—H9A	120.3	Ni1—O5—H5C	125 (3)
C8—C9—H9A	120.3	Ni1—O5—H5B	123 (3)
C11—C10—C9	117.1 (4)	H5C—O5—H5B	111 (3)
C11—C10—C13	121.2 (4)	Ni1—O6—H6B	121 (3)
C9—C10—C13	121.7 (4)	Ni1—O6—H6C	112 (5)
C10—C11—C12	120.0 (4)	H6B—O6—H6C	107 (3)
			
O2—C1—C2—N1	−175.7 (4)	O1—Ni1—N1—C2	1.3 (3)
O1—C1—C2—N1	3.1 (5)	O5—Ni1—N1—C6	90.5 (3)
O2—C1—C2—C3	2.9 (6)	N2—Ni1—N1—C6	7.3 (19)
O1—C1—C2—C3	−178.3 (4)	O6—Ni1—N1—C6	−92.5 (3)
N1—C2—C3—C4	0.0 (6)	O3—Ni1—N1—C6	−1.3 (3)
C1—C2—C3—C4	−178.5 (4)	O1—Ni1—N1—C6	179.4 (3)
C2—C3—C4—C5	−0.2 (7)	C11—C12—N2—C8	1.2 (7)
C3—C4—C5—C6	−0.1 (7)	C11—C12—N2—Ni1	178.3 (3)
C4—C5—C6—N1	0.7 (6)	C9—C8—N2—C12	−0.8 (7)
C4—C5—C6—C7	−179.1 (4)	C9—C8—N2—Ni1	−177.8 (3)
N1—C6—C7—O4	−177.3 (4)	N1—Ni1—N2—C12	4.0 (19)
C5—C6—C7—O4	2.5 (7)	O5—Ni1—N2—C12	−79.3 (3)
N1—C6—C7—O3	0.5 (5)	O6—Ni1—N2—C12	103.7 (3)
C5—C6—C7—O3	−179.7 (4)	O3—Ni1—N2—C12	12.5 (3)
N2—C8—C9—C10	0.1 (7)	O1—Ni1—N2—C12	−167.9 (3)
C8—C9—C10—C11	0.2 (7)	N1—Ni1—N2—C8	−179 (100)
C8—C9—C10—C13	−178.3 (4)	O5—Ni1—N2—C8	97.7 (4)
C9—C10—C11—C12	0.1 (7)	O6—Ni1—N2—C8	−79.3 (4)
C13—C10—C11—C12	178.6 (4)	O3—Ni1—N2—C8	−170.6 (3)
C10—C11—C12—N2	−0.8 (7)	O1—Ni1—N2—C8	9.0 (4)
C11—C10—C13—N4	170.7 (4)	N5—C14—N3—C13	−0.3 (5)
C9—C10—C13—N4	−10.8 (7)	C15—C14—N3—C13	177.4 (4)
C11—C10—C13—N3	−10.5 (7)	N4—C13—N3—C14	1.0 (5)
C9—C10—C13—N3	168.0 (4)	C10—C13—N3—C14	−177.8 (4)
N3—C14—C15—C19	8.3 (7)	N3—C13—N4—N5	−1.3 (5)
N5—C14—C15—C19	−174.3 (4)	C10—C13—N4—N5	177.6 (4)
N3—C14—C15—C16	−171.1 (5)	N3—C14—N5—N4	−0.5 (5)
N5—C14—C15—C16	6.3 (7)	C15—C14—N5—N4	−178.4 (4)
C19—C15—C16—C17	−1.3 (7)	C13—N4—N5—C14	1.1 (5)
C14—C15—C16—C17	178.1 (5)	C16—C17—N6—C18	0.8 (8)
C15—C16—C17—N6	0.3 (9)	C19—C18—N6—C17	−0.9 (7)
C16—C15—C19—C18	1.1 (7)	O2—C1—O1—Ni1	176.6 (3)
C14—C15—C19—C18	−178.2 (4)	C2—C1—O1—Ni1	−2.0 (4)
N6—C18—C19—C15	−0.1 (7)	N1—Ni1—O1—C1	0.5 (3)
C3—C2—N1—C6	0.6 (6)	O5—Ni1—O1—C1	90.7 (3)
C1—C2—N1—C6	179.3 (3)	N2—Ni1—O1—C1	179.9 (3)
C3—C2—N1—Ni1	178.6 (3)	O6—Ni1—O1—C1	−90.2 (3)
C1—C2—N1—Ni1	−2.7 (4)	O3—Ni1—O1—C1	−1.1 (5)
C5—C6—N1—C2	−1.0 (6)	O4—C7—O3—Ni1	176.1 (3)
C7—C6—N1—C2	178.8 (3)	C6—C7—O3—Ni1	−1.6 (4)
C5—C6—N1—Ni1	−178.9 (3)	N1—Ni1—O3—C7	1.6 (3)
C7—C6—N1—Ni1	0.9 (4)	O5—Ni1—O3—C7	−88.0 (3)
O5—Ni1—N1—C2	−87.5 (3)	N2—Ni1—O3—C7	−177.7 (3)
N2—Ni1—N1—C2	−170.7 (16)	O6—Ni1—O3—C7	91.6 (3)
O6—Ni1—N1—C2	89.5 (3)	O1—Ni1—O3—C7	3.2 (5)
O3—Ni1—N1—C2	−179.3 (3)		

### Hydrogen-bond geometry (Å, °)

**Table:**

*D*—H···*A*	*D*—H	H···*A*	*D*···*A*	*D*—H···*A*
N5—H5···O4^i^	0.88 (4)	1.81 (4)	2.685 (5)	179 (7)
O5—H5B···O1^ii^	0.86 (4)	1.87 (4)	2.718 (5)	168 (5)
O5—H5C···O2^iii^	0.86 (4)	1.89 (4)	2.705 (5)	160 (4)
O6—H6B···N6^iv^	0.87 (4)	1.93 (4)	2.781 (5)	167 (6)
O6—H6C···O3^v^	0.86 (4)	2.39 (4)	3.246 (5)	169 (8)
O6—H6C···O4^v^	0.86 (4)	2.41 (7)	3.081 (5)	135 (6)

Symmetry codes: (i) *x*+1/2, *y*+1/2, *z*; (ii) −*x*+1/2, −*y*+3/2, −*z*; (iii) *x*, *y*−1, *z*; (iv) −*x*+1, *y*+1, −*z*+1/2; (v) *x*, *y*+1, *z*.
